# Protocol on a systematic review of qualitative studies on asthma treatment challenges experienced in Sub-Saharan Africa

**DOI:** 10.1186/s13643-019-1068-7

**Published:** 2019-06-25

**Authors:** Pisirai Ndarukwa, Moses John Chimbari, Elopy N. Sibanda

**Affiliations:** 1 School of Nursing and Public Health, University of KwaZulu-Natal, 1st Floor, George Campbell Building, Howard College Campus, UKZN, Durban, 4000 South Africa; 2 Asthma, Allergy and Immunedsyfunction Clinic, 113 Kwame Nkrumah Ave, Harare, Zimbabwe

**Keywords:** Asthma, Treatment, Challenges, Experiences, Sub-Saharan Africa

## Abstract

**Background:**

Asthma is a major worldwide public health problem affecting an estimated 334 million people with over 300,000 deaths annually. Twenty-two million disability-adjusted life years (DALYs) are lost annually due to asthma. The condition may present many challenges if not managed well and effectively. This systematic review will provide a comprehensive synthesis of qualitative literature regarding the challenges experienced in the management of asthma and strategies adopted to counter these challenges. The review will answer the following questions: (i) what challenges have been experienced in the treatment of asthma in Sub-Saharan Africa (SSA)? and (ii) what strategies have been used to overcome asthma treatment challenges in SSA?

**Methods:**

The reviewers will search for the following databases for relevant qualitative studies: PubMed/MEDLINE, Scopus/Embase (Elsevier), EbscoHost, Cumulative Index to Nursing and Allied Health Literature (CINAHL) and Google Scholar, using the Medical Subject Headings (MeSH) and controlled vocabulary. These articles must have been published in the English language between January 2008 and December 2018. The identified papers will then be assessed for meeting eligibility criteria. Two independent reviewers will screen titles and abstracts of articles and then review the full texts of the selected research articles. Standard data extraction forms will be utilised, and the quality of the included studies will be assessed using the Joanna Briggs checklist for qualitative research appraisal tool. Results from eligible articles will be qualitatively synthesised using the framework synthesis approach and reported according to the Enhancing transparency in reporting the synthesis of qualitative research (ENTREQ) statement.

**Discussion:**

This systematic review will provide an overview of reported challenges in the treatment of asthma in Sub-Saharan Africa from 2008 to 2018. The review is expected to provide information that will help form the basis for future research, policy development and practice in treatment of asthma.

**Systematic review registration:**

PROSPERO CRD42018095802

**Electronic supplementary material:**

The online version of this article (10.1186/s13643-019-1068-7) contains supplementary material, which is available to authorized users.

## Background

Globally, asthma affects an estimated 334 million people and approximately over 300,000 deaths occur worldwide [1]. According to the Global Burden of Diseases (GBD) 2015 report on asthma, 22 million disability-adjusted life years (DALYs) are lost annually [2]. Asthma affects people across the lifespan, for instance, children with untreated asthma may miss much of their primary school education which leads to reduced educational opportunities and increased time off work for parents or guardians resulting in the negative impacts on the economy through loss of productivity [2].

Asthma is defined as a chronic inflammatory disease of the small airways [2]. Asthma remains an ailment with visibly unsatisfactory therapeutics with exacerbation of symptoms [3–5]. However, the evolvement of asthma treatment has over the decades, managed to minimise asthmatic attacks [6–8]. This has resulted in those suffering from the condition to have a good quality of life [8]. Despite these improvements in asthma treatment and worldwide distribution of asthma guidelines, most patients with asthma continue to experience some challenges in controlling asthma, especially in Sub-Saharan Africa (SSA) [9].

Several factors are reported to determine the challenges experienced by asthma patients in Sub-Saharan Africa [5, 10]. Air pollution, tobacco smoking and poor adherence to medications are some of the factors that are reported to affect asthma control in SSA [3, 8]. In addition, low levels of knowledge, negative attitudes and practices by asthmatic patients also remain as pertinent factors hindering access to quality asthma care. The quality of services is also an important determinant in deciding to utilise or achieve asthma treatment.

However, in the developed world, asthma treatment has had major significant improvements owing to availability of resources and treatment guidelines [11]. This has been noted to be in sharp contrast with most Sub-Saharan African countries which do not have asthma treatment guidelines with the exception of a few such as South Africa and Malawi [11]. Implementation of the treatment guidelines has been demonstrated to reduce morbidity and mortality of asthma [1].

There has been limited previous studies in SSA on treatment of asthma and no review to date has specifically focused on challenges of asthma treatment and solutions to these challenges. While some reviews have focused on asthma programmes in diverse regions of the world [12], these have not specifically focused on challenges relating to asthma treatment and solutions thereof to these challenges. The knowledge of these asthma treatment challenges will be useful in countries where treatment may not be easily available.

The challenges for treatment of asthma may adversely influence treatment outcomes for asthma patients and their quality of life [13]. There is, therefore, need to better understand the challenges to treatment of asthma patients in order to improve their quality of life. This will enable the creation of supportive environments for asthmatic patients which seek to encourage them to access treatment and services more readily. Through the proposed systematic review, we will collate challenges experienced on asthma treatment in SSA and strategies that have been employed to address these challenges. The findings will also be used as an input for decision-makers and policy formulators to plan and implement evidence-based strategies to solve challenges experienced in asthma treatment. Therefore, this systematic review will explore challenges experienced in asthma treatment and strategies that can be instituted to address these challenges. The study will set out to answer the following questions: (i) what challenges have been experienced in the treatment of asthma in SSA? and (ii) what strategies have been used to overcome asthma treatment challenges in SSA?

## Methods

In this systematic review, we will use the enhancing transparency in reporting the synthesis of qualitative research (ENTREQ) statement [14] which has been used for the preparation of this protocol. This protocol has been registered on the PROSPERO database (ref: CRD42018095802). PROSPERO was also searched to ensure a similar systematic review study protocol has not been registered. No prior studies of our topic of interest have been identified.

### Study design

A systematic review of the current evidence on challenges experienced in the treatment of asthma is considered a robust way of identifying and synthesising the peer reviewed articles for evidence in this area to define a cohesive empirical research agenda that builds on prior knowledge [15]. This review will include qualitative evidence only to produce an interpretation built on people’s views and experiences, acknowledging the rich context and different dimensions of the challenges from the perspective of those experiencing these challenges. Further, a synthesis of qualitative data aims to generate findings that are meaningful, relevant and appropriate to individuals, to inform a research agenda and ultimately to more effectively influence policy and practices on quality of care for asthma patients.

The review will use methods of qualitative synthesis to combine, integrate and interpret, where possible, the evidence from the included papers (see “Study eligibility criteria” and “Data synthesis and analysis” sections) [16, 17].

The review aims to move beyond the aggregation of available data to provide further interpretive insights into the challenges and strategies thereof to these challenges in the treatment of asthma and define where future research can add to what is known [17].

### Study eligibility criteria

The review will include qualitative peer-appraised studies. Qualitative data from mixed methods-studies, case studies, grounded theory, phenomenological studies and ethnographic methods will be screened for inclusion and included if the qualitative element is pertinent. Also, any study that uses qualitative methods for data collection such as interviews (individual and focus group), and qualitative methods for data analysis such as thematic analysis will be included. We will include those studies that have been conducted in Sub-Saharan African countries. All peer-reviewed articles published in English, reporting challenges faced in asthma treatment from patient, family, healthcare worker perspective and healthcare delivery system will be included. Additionally, studies will be included that describe how to overcome these challenges so as to improve the quality of treatment for asthma. To be included for the review, the studies should have been published from January 2008 up to December 2018 to ensure the currency of the work while enabling a broad view of the emerging issues to be identified. Initially, we wanted to focus on Southern African countries. However, during our initial search of studies, we noted that there were very few research articles which focused on Southern African countries. We thus have decided that the systematic review be widened to include African countries within the Sub-Sahara region.

### Study participants

The review will include all studies that report on the challenges of asthma treatment from the perspective of all patient categories (adults and children), family, health workers and healthcare policy-makers and other stakeholders that we will come across in the studies on challenges experienced in treatment of asthma and solutions thereof to these challenges in Sub-Saharan African countries. We define healthcare workers as recommended by the World Health Organisation (WHO) as all people involved in the actions whose primary intent is to enhance health [18].

### Type of interventions

To ensure applicability of our review, we will include studies that focus on those medical management and psychosocial interventions in the homes and community for asthma treatments such that challenges and solutions may be identified in the context of the SSA countries. We will include any qualitative study that explores these challenges experienced in treatment of asthma and those that report strategies addressing these challenges. We will apply a broad definition of the term challenges as ‘any factor or attribute which results in poor control of asthma’ [9].

### Types of outcome measures

The phenomena of interest in this review are challenges experienced in the treatment of asthma and the solutions to these challenges experienced in treatment of asthma by patients, families, healthcare workers and healthcare policy-makers.

### Study exclusion criteria

The reviewers will exclude any studies not available in English, conference abstracts, books or grey literature and editorial comments. Studies reporting only quantitative data (e.g. cross-sectional, case control, cohort studies and clinical trials) will be excluded.

### Search strategy

In collaboration with a medical librarian (MM), a systematic search strategy will be developed using a combination of Medical Subject Headings (MeSH) and controlled vocabulary to identify peer-reviewed articles on challenges experienced in asthma treatment and strategies thereof to these challenges (see Additional file 1). The databases will be PubMed/MEDLINE, Scopus/Embase (Elsevier), EbscoHost, CINAHL and Google Scholar. We will limit our search from January 2008 to December 2018 in order to capture challenges to asthma treatment and strategies to address these challenges. The care pathways are ever changing; hence, the need for reviews to be time factored to give a detailed account of what is contemporary [19].

#### Selection of study and process of data management

The ENTREQ guidelines [14] for reporting qualitative systematic reviews will be used to demonstrate the selection processes and results [14]. All retrieved studies will initially be imported into Endnote library to assist removing duplicates. After removing the duplicates, the Endnote library will be shared between the two reviewers (PN and MJC) to independently screen the articles by title and abstract, guided by the eligibility criteria. The studies which the two reviewers would have agreed on will be subjected to the full-text review. A third reviewer (ENS) will adjudicate any discrepancies between the two reviewers (PN and MJC). The two reviewers will independently review the full text of all eligible studies. In the case where there are differences between the two reviewers, consensus will be sought through discussion on the differences with the third reviewer. Finally, the full texts of all relevant studies found to meet the inclusion criteria will be retained for the final framework synthesis [20].

#### Quality appraisal

All retrieved articles eligible for inclusion will undergo a quality assessment process during the synthesis of results. The quality appraisal will be conducted by two independent reviewers (PN and MJC) using the Joanna Briggs Institute’s Critical Appraisal Checklist for Qualitative Research assessment tool (see Additional file 2) [21]. Where there are disagreements between the two reviewers, a third reviewer (ENS) will be engaged and discussions among the three reviewers will be used to resolve the differences. This tool was developed primarily for use in systematic reviews. The results will help us to determine if studies included are consistent with the standard quality appraisal for articles reporting on qualitative studies.

#### Data extraction

Data will be independently extracted by two review authors (PN and MJC) from eligible studies onto a customised data extraction form and populated with variables pertaining to the study population and phenomena of interest. Double checking and verification of extracted articles will be done by the third review author (ENS). The reviewers will make use of an adapted Joanna Briggs Institute (JBI) data abstraction format [21] (see Additional file 3). Study characteristics that will be extracted will include name of the first author and year of publication, data collection period and country in which the study was conducted. Specific study details including the study design, study population, sample size, sampling procedures and data collection procedures will then be captured. Factors reported as challenges/problems/problem issue(s) to asthma treatment and strategies to these challenges will be systematically identified.

#### Data synthesis and analysis

In an attempt to demonstrate differences among subgroups such as patients, families, healthcare workers and healthcare policy-makers, the researchers will analyse and report findings from these clusters separately. The thematic framework analysis approach will be adopted in the analysis and synthesis of data [20]. Thematic synthesis is worthwhile where the evidence is likely to be largely descriptive and will improve our appreciation of the challenges of asthma treatment and solutions thereof to these challenges. We will follow the five stages of framework synthesis to synthesise our qualitative data.

##### Familiarisation with the data

The first reviewer (PN) will begin with familiarisation of the data against the aims of the review and note recurrent themes across the studies.

##### Identifying a thematic framework

The reviewers will use a pre-determined thematic framework which was developed by the review authors using literature (see Additional file 4) to guide the thematic analysis instead of developing own priori framework. However, our review will adopt this framework based on the emerging themes from our analysis. This framework offers a detailed list of likely factors that could contribute to challenges and or possible strategies used to overcome these challenges in asthma treatment.

##### Indexing

The two reviewers (PN and MJC) will independently read the extracted information to search for themes according to a predetermined thematic framework and additional emergent themes. The framework will be revised as new themes emerge. This will be done through dialogue and agreement by the entire review team. All studies will be read until there are no new emerging themes. Coding of the data will be done based on the themes identified in the data. Each primary study will be indexed using the codes related to the themes of the framework. Where appropriate, parts of the studies may be indexed with one or more codes.

##### Charting

The reviewers will sort the data by theme and present the themes in the form of an analysis table (chart). The columns and rows of the table will reflect the studies, and related themes and will enable us to compare findings of the studies across different themes and subthemes.

##### Mapping and interpretation

The reviewers will use charts to define the identified concepts and map the range and nature of the phenomena. Our review will explore associations between the themes to help clarify the findings. Our review will map and interpret findings in line with the review objectives and emerging themes.

## Discussion

Asthma treatment in low-resourced settings such as Sub-Saharan Africa is often hampered by unavailability and non-adherence to international asthma management guidelines. Most countries in SSA have weakened public health systems which makes it difficult to adopt multisectoral approaches to asthma management. This systematic review will contribute to what is known, giving novel attention to challenges and strategies to address these challenges. This is considered a necessary step in the quality of care for asthma research. Building on the knowledge base will contribute to a better understanding of the complex challenges experienced in the treatment of asthma and the push towards improving the quality of asthma care. While these theoretical insights to a subject area can add value to the academic evidence base, it is important to produce a robust qualitative synthesis that will reflect on the identified evidence. A framework synthesis can adequately meet the proposed objectives, provide answers to research questions and speak meaningfully to policy directives. The results from the review will inform and guide healthcare practitioners and researchers on developing appropriate and feasible interventions aimed at enhancing the treatment of asthma in resource-constrained settings.

### Ethics and dissemination

The reviewers did not seek ethical approval for conducting the systematic review as it will not involve access to individual-level data. The study was registered with the PROSPERO under registration number CRD42018095802. Findings from this systematic review will be communicated to a broad range of stakeholders. We will also publish the systematic review paper in accredited journals. To ensure uptake of the review results, reviewers will also engage with the policy-makers and research funders to ensure utilisation of the results of the systematic review.

## Additional files

Additional file 1: Search strategy. (DOCX 12kb)

Additional file 2: JBI quality appraisal tool. (DOCX 110 kb)

Additional file 3: JBI data extraction tool. (DOCX 57 kb)

Additional file 4: Thematic framework analysis format. (DOCX 11 kb)

## Abbreviations

CINAHL: Cumulative Index to Nursing and Allied Health Literature
ENTREQ: Enhancing transparency in reporting the synthesis of qualitative research
JBI: Joanna Briggs Institute
PROSPERO: International Prospective Register of Systematic Reviews
SSA: Sub-Saharan Africa
UKZN: University of KwaZulu-Natal
WHO: World Health Organisation
