# Supplementary material for: Protocol on a systematic review of qualitative studies on asthma treatment challenges experienced in Sub-Saharan Africa
Source: Syst Rev. 2019 Jun 25;8:149. doi: 10.1186/s13643-019-1068-7 (PMC6593567; doi:10.1186/s13643-019-1068-7)
Supplement: Supplementary file 1 — Search strategy. (DOCX 12kb) [file 13643_2019_1068_MOESM1_ESM.docx]

**Table 1 Search strategy**

| **Key word** | **Alternative word** |
| --- | --- |
| Qualitative studies | Qualitative studies OR Case studies OR Focused group discussions OR phenomenological studies OR grounded theory studies OR ethnographic studies OR interviews |
| Challenges | Challenges OR Challenge OR Problem OR Problems barriers or Difficulties or Issues or Limitations or Obstacles |
| Treatment | "Asthma/therapy"[Mesh]OR "Therapeutics"[Mesh] OR Care OR Therapeutic OR Therapy OR Therapies OR Treatment OR Treatments OR Intervention |
| Asthma | "Asthma"[Mesh] OR Asthmas OR Bronchial Asthma OR Asthma, Bronchial |
| Sub-Saharan Africa | Africa filter: Angola OR Benin OR Botswana OR Burkina Faso OR Burundi OR Cameroon OR Cape Verde OR Central African Republic OR Chad OR Comoros OR Congo OR Democratic Republic of Congo OR Djibouti OR Equatorial Guinea OR Eritrea OR Ethiopia OR Gabon OR Gambia OR Ghana OR Guinea OR Guinea Bissau OR Ivory Coast OR Cote d’Ivoire OR Kenya OR Lesotho OR Liberia OR Madagascar OR Malawi OR Mali OR Mauritania OR Mauritius OR Mozambique OR Namibia OR Niger OR Nigeria OR Principe OR Reunion OR Rwanda OR Sao Tome OR Senegal OR Seychelles OR Sierra Leone OR Somalia OR South Africa OR Sudan OR Swaziland OR Tanzania OR Togo OR Uganda OR Western Sahara OR Zambia OR Zimbabwe OR Central African OR West Africa OR Western African OR Eastern African OR South African OR Southern Africa OR sub Saharan Africa |
