# Supplementary figures and images for: Protocol on a systematic review of qualitative studies on asthma treatment challenges experienced in Sub-Saharan Africa
Source: Syst Rev. 2019 Jun 25;8:149. doi: 10.1186/s13643-019-1068-7 (PMC6593567; doi:10.1186/s13643-019-1068-7)

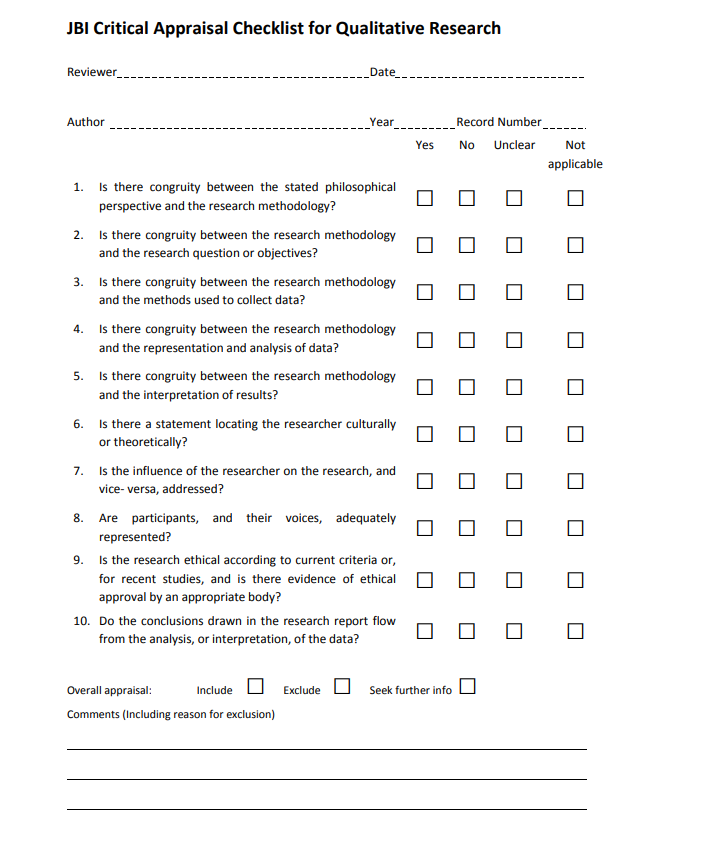


Copyright © The Joanna Briggs Institute 2014

Supplement: Supplementary file 2 — JBI quality appraisal tool. (DOCX 110 kb) [file 13643_2019_1068_MOESM2_ESM.docx]

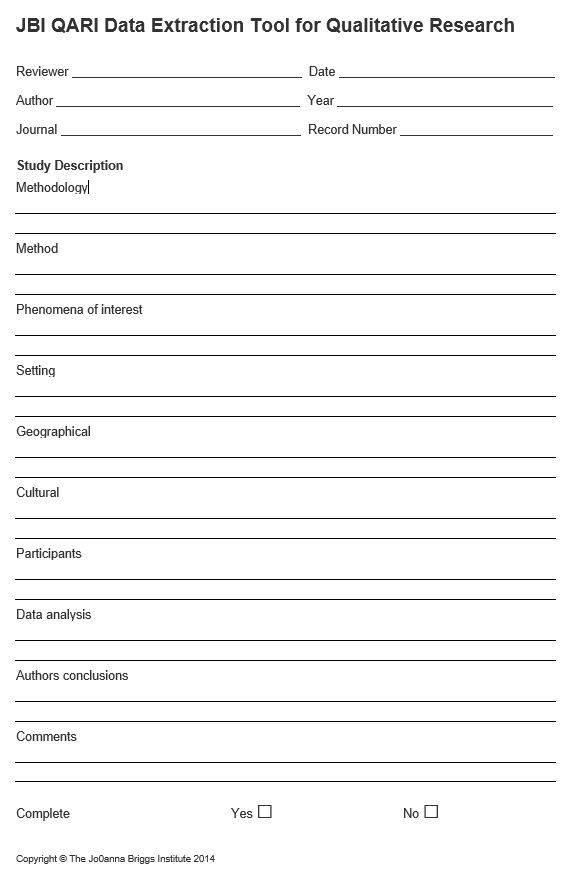


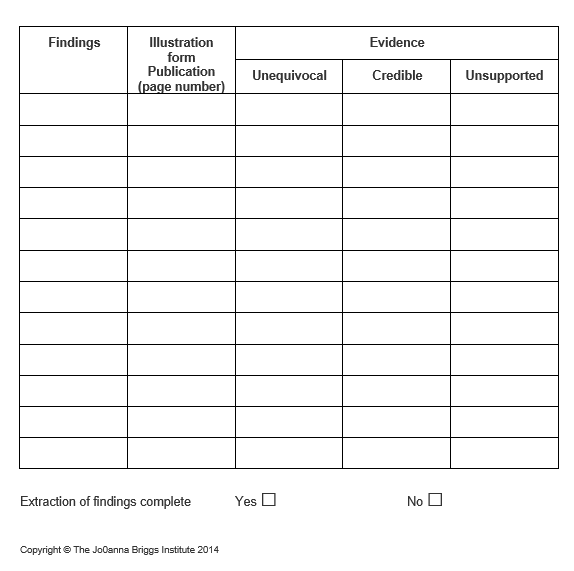

Supplement: Supplementary file 3 — JBI data extraction tool. (DOCX 57 kb) [file 13643_2019_1068_MOESM3_ESM.docx]
