# Supplementary material for: Protocol on a systematic review of qualitative studies on asthma treatment challenges experienced in Sub-Saharan Africa
Source: Syst Rev. 2019 Jun 25;8:149. doi: 10.1186/s13643-019-1068-7 (PMC6593567; doi:10.1186/s13643-019-1068-7)
Supplement: Supplementary file 4 — Thematic framework analysis format. (DOCX 11 kb) [file 13643_2019_1068_MOESM4_ESM.docx]

**Thematic framework analysis format for summarizing asthma treatment challenges experienced in SSA.**

| **Main theme** | **Emerging sub-themes** | **Asthma treatment challenges** | **Studies** |
| --- | --- | --- | --- |
|  |  |  |  |
|  |  |  |  |
|  |  |  |  |
|  |  |  |  |
|  |  |  |  |
